# Spider Bite-Induced Facial Nerve Palsy

**DOI:** 10.7759/cureus.32162

**Published:** 2022-12-03

**Authors:** Laurence Stolzenberg, Alexis Koch, Austin Huang, Mohammad Usman, Shivani Subhedar, Trevor Decker, Raphael Quansah

**Affiliations:** 1 Orthopedic Surgery, Alabama College of Osteopathic Medicine, Dothan, USA; 2 Internal Medicine, Alabama College of Osteopathic Medicine, Dothan, USA; 3 Neurology, Alabama College of Osteopathic Medicine, Dothan, USA; 4 Anesthesiology, Alabama College of Osteopathic Medicine, Dothan, USA; 5 Radiology, Alabama College of Osteopathic Medicine, Dothan, USA; 6 Internal Medicine, Decatur Morgan Hospital, Decatur, USA

**Keywords:** bell's palsy, loxoscelism, facial nerve paralysis, facial nerve palsy, brown recluse, septic shock

## Abstract

Spider bites, while rarely confirmed beyond a doubt, should always be in the differential for any severe symptoms or infection out of proportion to presentation with the suspected presence of appropriate vectors. While most arthropod bites will only result in mild localized irritation, the potential to cause severe cutaneous and systemic effects should not be overlooked. We present one such case, in which a presumed brown recluse (*Loxosceles reclusa*) bite on the neck resulted in severe illness with systemic manifestations. The patient presented to the emergency room minimally responsive with left-sided facial nerve palsy and septic shock. While the admitting physician initially prioritized stabilizing the patient, he noted the left-sided cervical cellulitis. Thorough history taking revealed that the patient had been worsening since being bitten by a spider three days prior to admission. After a month-long hospital stay and multidisciplinary treatment, the patient was transferred to a larger center with facial paralysis still present.

## Introduction

Facial nerve palsy is an acute paralysis of the muscles innervated by the seventh cranial nerve. This condition can present with hemiplegia or paralysis on both sides of the face but more often presents on only one side. Facial nerve palsy may be complete or partial, where only some or all facial muscles are affected on one side [1]. The condition is usually characterized by a lower motor neuron pattern [1]. Patients with this condition often present with dry eyes and an isolated facial paralysis of the forehead and other facial muscles [2]. Other associated symptoms are numbness, mild pain, hyperacusis, and dysgeusia [1,2]. The presentation is usually quite rapid, progressing to the expected clinical picture over one to three days [2]. The most common complication reported in the literature was incomplete eyelid closure causing dry eye. The most severe complication linked to facial palsy was varying levels of permanent facial paralysis and muscle contractures [2].

The epidemiology of facial nerve palsy shows a higher incidence in people aged 15-40 years old, with the peak being closer to 40 years old [2]. It is more common in diabetic patients [2]. The hypothesized etiology is inflammation resulting in the compression of the seventh cranial nerve and is widely accepted as the underlying cause of the paralysis. This can lead to ischemia due to the interruption of the nerve’s blood supply ultimately resulting in demyelination. The most common underlying cause of this inflammation is idiopathic [2], although many cases have been linked to herpes simplex virus type 1 (HSV-1) as a probable cause [1]. Other potential etiologies are numerous such as malignancy, trauma, hypertension, and Lyme disease, caused by *Borrelia burgdorferi* [2,3].

The prognosis of this condition is good, although multiple factors have been linked with a poor outcome. These include a complete palsy, no improvement in symptoms within three weeks, age greater than 50 years, and numerous others [4]. Treatment will vary widely based on the underlying cause, although most often clinicians administer steroids, such as prednisolone. Viral causes often require antivirals such as acyclovir or valacyclovir [5], which may be given empirically in idiopathic cases. Palsy due to trauma may require surgical intervention.

While an association with the *Ixodes scapularis* tick is well-known, an in-depth literature review revealed no cases of spider bites inducing facial nerve palsy. A review did however reveal one report of transverse myelitis and partial paralysis due to a bite from *Loxosceles reclusa* [6]. There was also a case of partial paralysis due to a *Latrodectus mactans* bite [7]. It is therefore sufficient to say that paralysis caused by spider bites is not completely unheard of, although it appears to be very uncommon with few published cases.

The brown recluse is a common arachnid in the United States [7]. This spider's bite causes a condition known as loxoscelism, also called "gangrenous spot" [7]. As implied by the previously mentioned name, the bite can cause full-thickness skin necrosis, although it usually causes only local erythema and pain [7]. Systemic manifestations include autoimmune hemolysis and disseminated intravascular coagulation (DIC), and even death is a possibility but is uncommon [7]. The severity of the reaction depends on the amount of venom transferred and the immune status of the victim. The bite of this spider is considered quite common in the United States [7]. Diagnosis is often clinical, based on presentation and history. Treatment varies widely based on presentation and symptoms, ranging from local pain control to frequent laboratory testing and treatment for autoimmune hemolytic anemia and DIC.

## Case presentation

The patient was a 64-year-old Caucasian female with underlying medical conditions including a history of hepatitis C virus (HCV) infection, diabetes mellitus (DM), a history of myocardial infarction (MI), and the placement of a pacemaker. According to the family, the patient lives in a rural part of Alabama near fields and wooded areas. She was brought to the emergency department (ED) by her family for left-sided facial drooping. They suspected that she may have overdosed on her prescribed muscle relaxant, tizanidine. On physical examination, the emergency physician noticed cellulitis on her neck and a fractured left thumb. Initial treatment was aimed at the possible overdose, while imagery was being taken, and laboratory tests were run. The patient was initially hypotensive with a blood pressure of 73/46 mmHg. She was treated empirically with 3 L of intravenous (IV) fluids, norepinephrine, dexamethasone, diphenhydramine for possible extrapyramidal symptoms, and epinephrine.

**Figure 1:**
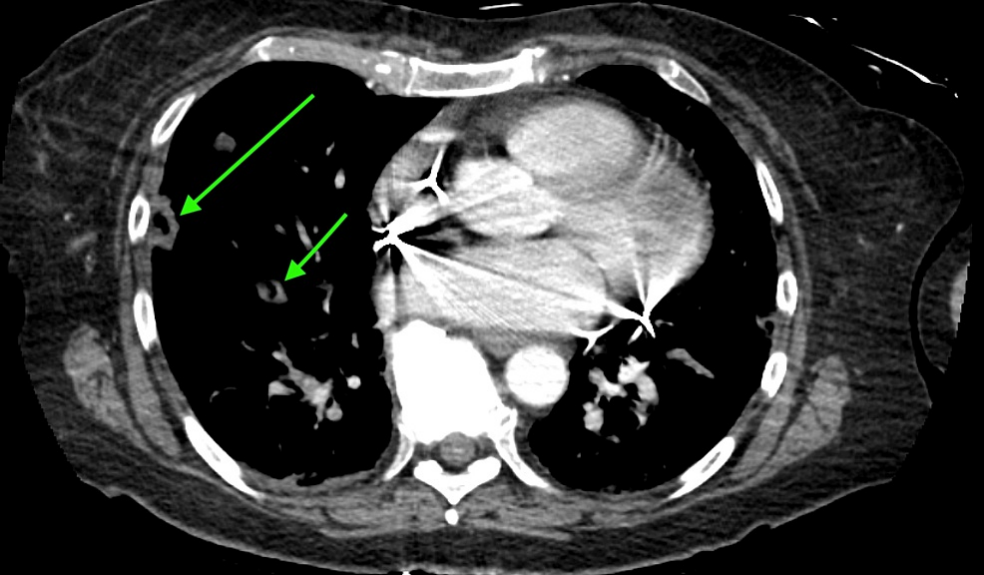
Lung CT, transverse cut, with cavitation suggestive of septic pulmonary emboli. CT: computed tomography

**Figure 2:**
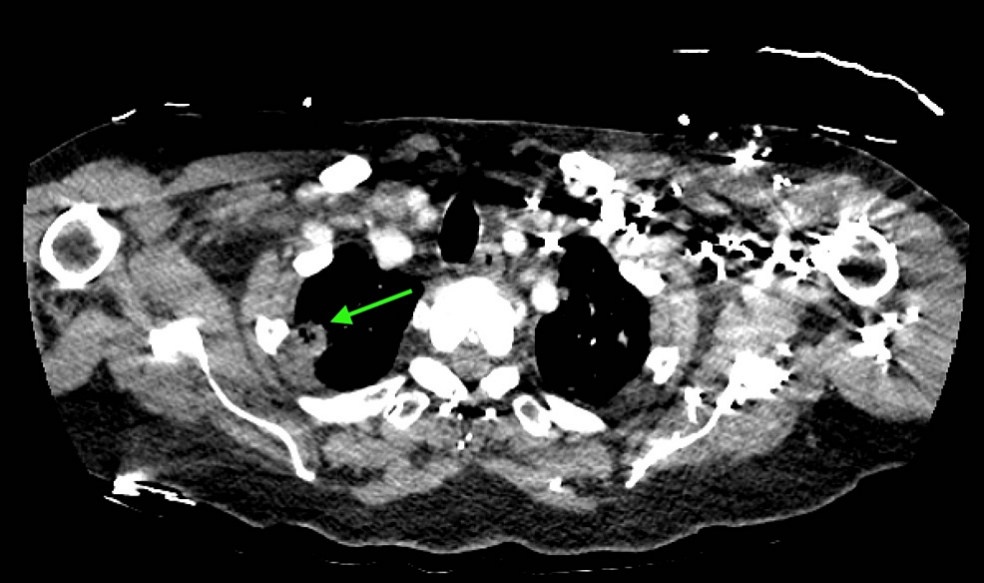
Apical lung CT, transverse cut, with cavitation suggestive of septic pulmonary emboli. CT: computed tomography

**Figure 3:**
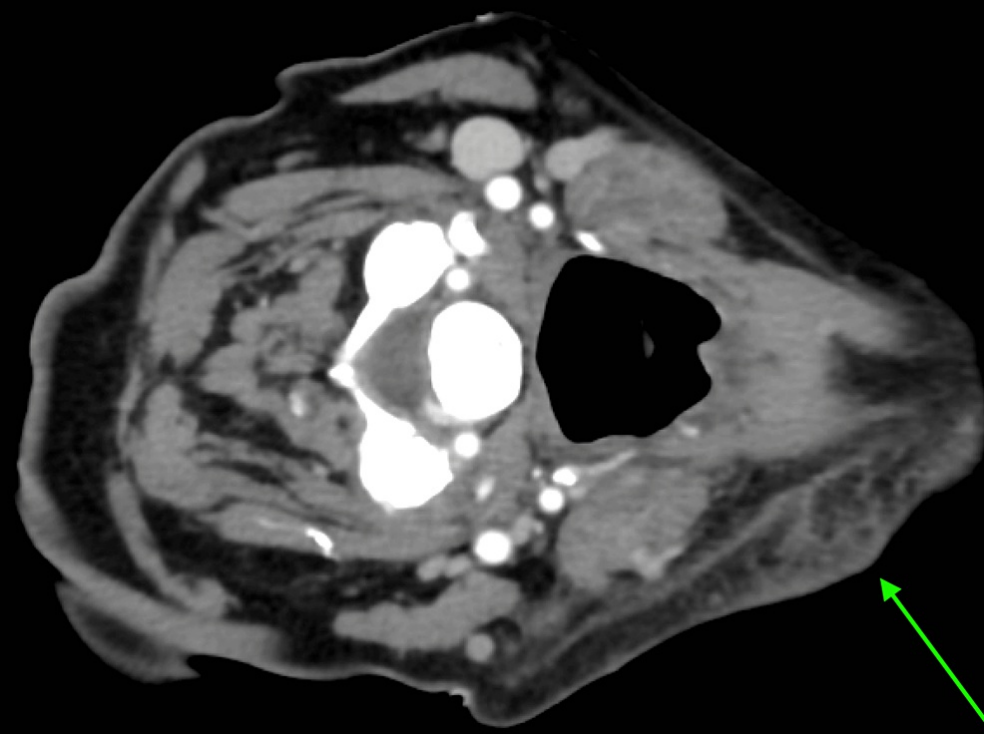
Neck CT, transverse cut, showing cellulitis (rotated 90 degrees clockwise). CT: computed tomography

Initial imaging included a negative chest X-ray, a head computed tomography (CT) that showed only chronic microvascular changes, a hand X-ray showing a proximal phalangeal fracture, and an abdominal/pelvic CT that showed no abnormalities. Finally, an upper chest CT showed "septic emboli with cavitary nodular changes throughout the lungs bilaterally and no large or visible pulmonary emboli" (Figures 1-2). Additionally, a neck CT confirmed cellulitis in the neck (Figure 3). Based on this examination, the following preliminary diagnoses were made: septic shock, septic embolism, cellulitis, and closed fracture of the left thumb. The emergency physician consulted a hospitalist for admission and further management.

Upon inpatient admission, further questioning revealed that the patient had been bitten three days ago on the left side of her neck. The family was unsure what exactly bit her, but the patient thought it was a spider. Her family also confirmed that they had seen many brown recluse spiders in their home. The family endorsed that her neck had been gradually getting more swollen and erythematous and that she was becoming gradually more obtunded. Following treatment in the emergency department, her blood pressure had also risen to 104/47 mmHg, her pulse was 60 beats per minute, her respiration rate was of 19, and her temperature was 97.3°F. Physical examination revealed a partially oriented female lying in bed who could follow basic commands but was not oriented to time. The examination of her neck showed erythema in the submandibular region going over the mandible with palpable lymph streak down to the clavicle on the left side. The examining physician also noticed a central area with tender nodulations and noted a lack of fluctuations. The cardiovascular, pulmonary, and gastrointestinal examinations were unrevealing. The examination of the head and neck revealed left-sided facial nerve palsy in a lower motor neuron pattern.

Her complete metabolic panel (CMP) was notable for a sodium of 133 mEq/L, a potassium of 3.4 mEq/L, and a chloride of 93 mEq/L. Her complete blood count (CBC) was notable for a white blood cell count of 12,000 cells/μL (87.4% of which were neutrophils), a hemoglobin of 8.0 g/dL, and a hematocrit of 24.3%. Clinicians also found an elevated D-dimer (11.6 mg/L fibrinogen equivalent units). Renal function tests (RFTs) revealed a serum creatinine (SCr) of 1.3 mg/dL, a blood urea nitrogen (BUN) of 30 mg/dL, and an estimated growth factor receptor (GFR) of 41 ml/minute/1.73 m^2^, significantly below her previous baseline recorded three months prior. Results were also notable for elevated creatine kinase of 14,853 U/L suggesting rhabdomyolysis, elevated liver enzymes including aspartate aminotransferase (AST) of 478 U/L and alanine aminotransferase (ALT) of 128 U/L, and finally an elevated pro-brain natriuretic peptide (19,342 pg/ml). The workup included laboratory tests to confirm her HCV infection. She had a reactive HCV antibody test but negative quantitative HCV ribonucleic acid (RNA) test. She also had a hemoglobin A1C of 6.9%, showing relatively well-controlled DM, although her glucose at admission was 264 mg/dL. The workup also showed gram-positive cocci in two blood cultures.

The patient was admitted to the intensive care unit (ICU) for a higher level of treatment. The patient was empirically started on piperacillin/tazobactam and vancomycin for cellulitis and pulmonary emboli until an infectious disease consultation could occur. Infectious disease was consulted to evaluate the patient, at which point she was now obtunded and unresponsive. Later during her clinical course, the completed culture had revealed methicillin-resistant *Staphylococcus aureus* (MRSA), sensitive to vancomycin, trimethoprim-sulfamethoxazole (TMP-SMX), clindamycin, and gentamycin. It was only on the third day of this patient's stay that her facial nerve palsy was addressed. She had been considered critically ill, and stabilization was the priority. Multiple sequential maxillofacial and head CT scans were completed, and all showed extensive cellulitis and a lack of acute intracranial pathology with no other abnormalities. The patient had already been placed on steroids at this point for other issues, which is the most common treatment indicated for this pathology. They considered obtaining magnetic resonance imagery (MRI), but this was impossible due to the patients' pacemaker. Vascular surgery was consulted due to a concern of thrombophlebitis being linked to the new-onset facial droop. The surgeon ordered a computed tomography angiography (CTA) of the neck, which showed a new-onset internal jugular (IJ) deep vein thrombosis (DVT), but also ruled out any serious issues with the carotid arteries on either side. Following a suggestion for long-term anticoagulation, vascular surgery signed off from this case.

On the eighth day of hospitalization, the hospitalist group decided to consult neurology for the facial nerve paralysis that did not seem to improve with IV steroids. Neurology requested more imagery, all showing slowly resolving cellulitis and no further pathology. Their final recommendation was facial nerve stimulation as an outpatient following the resolution of septic shock and the numerous other conditions being treated. The patient's family was made aware of this suggestion.

On the 10th day of hospitalization, it was decided that steroids, initially methylprednisolone 40 mg IV once daily and briefly transitioned to prednisone 40 mg oral once daily, were bringing no additional benefit and were halted. The consensus among the care team was that the underlying cause was linked to the ongoing gram-positive bacteremia and severe cellulitis. These conditions were still being treated by intensive IV antibiotics and management by infectious disease physicians. Following this, her general condition worsened significantly due to acute hypoxemic respiratory failure, and in the following days, she was put on bilevel positive airway pressure (BiPAP) with 100% oxygen with numerous attempts to wean her.

At this point, her care was again focused on keeping her alive and stabilizing her. There was only a brief mention of the facial nerve paralysis, showing that the hospitalists were aware of it. On the 32nd day of her hospitalization, she was transferred to a university hospital capable of providing higher-level care. Her facial nerve palsy was still unresolved at that time. She was then lost to follow-up by the authors of this study.

## Discussion

This patient experienced an extensive and severe clinical course. She began in the intensive care unit (ICU) of a mid-sized hospital, where she remained in critical condition for over a month before being transferred to a university hospital able to provide a higher level of care. It is therefore reasonable to emphasize the importance for future patient management to study both the initial cause of her illness, determine if anything could have been done to better treat her, and consider this less typical association between facial nerve palsy and loxoscelism.

The first topic to discuss is the etiology of her illness. Although the physicians tentatively diagnosed the origin of her constellation of symptoms as being loxoscelism, no absolute confirmatory test exists [8,9]. Available literature mentions that the final diagnosis of loxoscelism required the recovery of the actual spider for proper identification by an entomologist [9], although this appears improbable in a real-world situation, and was certainly not the case here. In addition to the patient's and her families' recounting of the events, we can follow guidelines for the diagnosis of this condition. The literature suggests multiple criteria for the condition including the following: The patient presents with a single bite versus multiple, and the bite usually occurs in a more secluded setting such as the inside of a home [9,10]. There are also mentions that bites are usually flat, occur between March and November, ulcerate late, and are rarely, if ever, exudative [9,10]. All these criteria were met, which helped confirm the tentative diagnosis. Additionally, the literature reveals that there exists an enzyme-linked immunosorbent assay (ELISA) for the venom, but it does not appear to be available to clinicians, and the duration during which it is useful is not yet confirmed [8].

Having a good case to affirm the etiology of her presentation, we can discuss what the literature recommends for treating brown recluse bites. Based on existing literature, the general guidelines were followed except for one main intervention that was not considered, that is, surgical debridement [11]. Further review of the case shows that for this patient, it may not have been useful. This is due to the assessment by clinicians that the major pathophysiological process at play was the dissemination infection, which could already be observed at admission. Based on this case, it stands to reason that the best way to prevent other similar cases would be to ensure better patient education on recognizing loxoscelism and associated complications to facilitate earlier intervention. Additionally, the literature suggests the use of sonography for the early detection of severe infection, notably necrotizing fasciitis, secondary to cutaneous loxoscelism [12]. However, again, this intervention would have required the patient to present earlier for any advantage.

As for the facial paralysis, as previously discussed, the most common etiology of facial nerve palsy is idiopathic [2]. Many physicians will have this condition, and the treatments associated with it, as the first item in their differentials when observing this presentation. Although our case was obviously complicated by significant systemic symptoms pointing toward infection, in less obvious cases, other etiologies such as a bacterial infection causing inflammation through different mechanisms may not make it on a physician's differential. This distinction is important, as treating the underlying infection takes precedence over treating the inflammation itself. So, antibiotics and debridement as needed would be a reasonable first step. Whichever the case, the use of intravenous steroids is useful for both idiopathic cases of palsy and facial paralysis associated with infection and sepsis [13,14]. It is nevertheless important to mention that in this case, the use of intravenous steroids resulted in no clear improvement based on available information. Finally, one more promising new therapy for this condition is low-level laser therapy (LLLT), which could be used if ever the patient is properly stabilized [15].

The last learning point is the overall unusual presentation of loxoscelism with facial nerve palsy as one of the first things noticed by the clinicians. Considering this patient's clinical deterioration and prolonged hospitalization, any sign preceding serious bacteremia and septic shock in similar cases can give physicians advance warning and the opportunity to treat these serious conditions early, which is crucial for the prognosis of this condition [16]. It is therefore particularly important to keep this link in mind when evaluating otherwise unimpressive integumentary symptoms and facial nerve paralysis of unknown etiology.

## Conclusions

We hope that this unique case of facial nerve palsy associated with loxoscelism highlights a rare potential cause of seventh cranial nerve paralysis for clinicians to keep in their differential. The importance of keeping rare but potentially deadly etiologies in mind is of vital importance to the management of any acutely ill patient. Certainly, spider bites rarely cause such severe symptoms, but since so much of the population of the United States is at risk of exposure, it is critical to keep it in the differential. As we have already outlined, determining the etiology of the facial nerve paralysis plays a key role in the appropriate management of this condition, as well as the prevention of significant complications that may arise. Notably though, correctly diagnosing loxoscelism is certainly a challenge due to the difficulty in capturing the original vector and the lack of absolute confirmatory testing, so reviewing different rare presentations may aid in confirming any clinical suspicion.
